# Intimate partner and client‐perpetrated violence are associated with reduced HIV pre‐exposure prophylaxis (PrEP) uptake, depression and generalized anxiety in a cross‐sectional study of female sex workers from Nairobi, Kenya

**DOI:** 10.1002/jia2.25711

**Published:** 2021-06-24

**Authors:** Maria Leis, Miranda McDermott, Alex Koziarz, Leah Szadkowski, Antony Kariri, Tara S Beattie, Rupert Kaul, Joshua Kimani

**Affiliations:** ^1^ Department of Medicine University of Toronto Toronto Canada; ^2^ Biostatistics Research Unit University Health Network Toronto Canada; ^3^ Department of Medical Microbiology University of Nairobi Nairobi Kenya; ^4^ Department of Global Health and Development London School of Hygiene and Tropical Medicine London England; ^5^ Department of Medical Microbiology University of Manitoba Manitoba Canada

**Keywords:** female sex workers, HIV, intimate partner violence, depression, anxiety, pre‐exposure prophylaxis

## Abstract

**Introduction:**

UNAIDS has identified female sex workers (FSW) as a key HIV at‐risk population. FSW disproportionately experience gender‐based violence, which compounds their risk of HIV acquisition and may contribute to adverse mental health outcomes. Pre‐exposure prophylaxis (PrEP) is a powerful but underused HIV prevention tool for these women. This study explored the associations between intimate partner violence (IPV) and client‐perpetrated violence against FSW, mental health outcomes and PrEP use.

**Methods:**

An anonymous questionnaire was administered to a convenience sample of 220 Nairobi FSW attending dedicated clinics from June to July 2019, where PrEP was available free of charge. A modified version of the WHO Violence Against Women Instrument assessed IPV and client‐perpetrated violence, and the Patient Health Questionnaire‐9 (PHQ‐9) and Generalized Anxiety Disorder‐7 (GAD‐7) assessed depressive and anxiety symptoms respectively. Multivariable logistic regressions evaluated predictors of depression, generalized anxiety and PrEP use.

**Results:**

Of the total 220 women (median [IQR] age 32 [27‐39]), 56.8% (125/220) reported depression (PHQ‐9 ≥ 10) and 39.1% (86/220) reported anxiety (GAD‐7 ≥ 10). Only 41.4% (91/220) reported optimal use of PrEP (taken correctly six to seven days/week) despite the cohort pursuing sex work for a median of 7 (4 to 12) years. Most women reported experiencing any violence in the past 12 months (90%, 198/220). Any recent IPV was frequent (78.7%, 129/164), particularly emotional IPV (66.5%, 109/164), as was any client‐perpetrated violence in the past 12 months (80.9%, 178/220). Regression analyses found that violence was independently associated with depression (adjusted OR [aOR] 9.39, 95% CI 2.90 to 30.42, *p* = 0.0002) and generalized anxiety (aOR 3.47, 95% CI 1.10 to 10.88, *p* = 0.03), with the strongest associations between emotional IPV and both depression and anxiety. Recent client‐perpetrated emotional violence (aOR 0.23, 95% CI 0.07 to 0.71, *p* = 0.01) was associated with decreased PrEP use, whereas client‐perpetrated physical violence was associated with increased PrEP use (aOR 3.01, 95% CI 1.16 to 7.81, *p* = 0.02).

**Conclusions:**

There was a high prevalence of recent violence by different perpetrators as well as depression and anxiety among FSW from Nairobi. PrEP use was relatively infrequent, and recent client‐perpetrated emotional violence was associated with PrEP non‐use. Interventions to reduce gender‐based violence may independently enhance HIV prevention and reduce the mental health burden in this community.

## 1 Introduction

There have been substantial declines in global HIV/AIDS incidence since the peak of the epidemic in 1998, and UNAIDS estimates that new HIV infections have been reduced by 40% [1]. However, while these decreases constitute important advances in the HIV response, they may mask sustained or expanding spread among key populations who are disproportionately affected by HIV [2]. Two‐thirds of the estimated 37 million people worldwide living with HIV/AIDS reside in sub‐Saharan Africa, with Kenya currently experiencing the third largest HIV epidemic in the world [3]. Women and girls living in sub‐Saharan Africa are more likely to be infected than men and boys [1, 4]. In particular, female sex workers (FSW) represent a key vulnerable population [1]. In Kenya, the overall prevalence of HIV in adults is 4.5 per 100 people; the prevalence of HIV in sex workers is 29.3 [3].

UNAIDS reports that more than one‐third of women around the world have experienced physical and/or sexual violence at some time in their lives [1]. In some regions, women who experience violence are one and a half times more likely to become infected with HIV [1], particularly for women in sub‐Saharan Africa [5]. Furthermore, violence is a common problem for women who sell sex [6]. A qualitative enquiry into experiences of violence for FSW in Kenya found that sexual and physical violence were pervasive, underscored by the extreme financial needs of FSW, gender‐power differentials, illegality of trading in sex and cultural subscriptions to men’s entitlement for sex without payment [7]. Further compounding HIV risk in this context is the fact that men who are violent towards women are more likely to have HIV [8]. Therefore, it is imperative to ensure access to effective HIV prevention methods as part of comprehensive violence prevention and response services for FSW populations.

Pre‐exposure prophylaxis (PrEP) is a powerful method of HIV prevention [9, 10]. Currently approved PrEP regimens consist of daily oral emtricitabine‐tenofovir, which has been demonstrated to significantly reduce HIV acquisition among high‐risk men who have sex with men, persons who use intravenous drugs and heterosexual men and women [11]. Among FSW, this may enable women to access an HIV prevention strategy that does not require disclosure to partners [12]. Additionally, women with a history of intimate partner violence (IPV) reported that they would be more willing to use PrEP compared to women without these experiences [13, 14], further underscoring the importance of HIV prevention in at‐risk populations. However, IPV is also associated with depression [15, 16] and substance use among FSW [17]. As such, mental health symptomology may contribute to challenges with PrEP adherence, as has been shown with other antiretroviral medication adherence [18, 19, 20]. Furthermore, different patterns of violence have been shown to be differentially associated with sexual risk outcomes [21]; following, those who perceive themselves to be at higher risk of HIV acquisition (i.e. physical, sexual violence) may have increased PrEP adherence compared to those at lower perceived immediate risk (i.e. emotional violence) [22]. Research has demonstrated that violence and emotional manipulation from sexual partners specifically heightens FSW HIV risk through engagement in higher risk sexual behaviours (i.e. less condom use, more partners) [23]. Similarly, FSW often have lower perceived control in these emotional relationships, with abuse in these scenarios being linked to increased HIV risk behaviours, which may contribute to reduced PrEP uptake [23, 24, 25].

**Figure 1:**
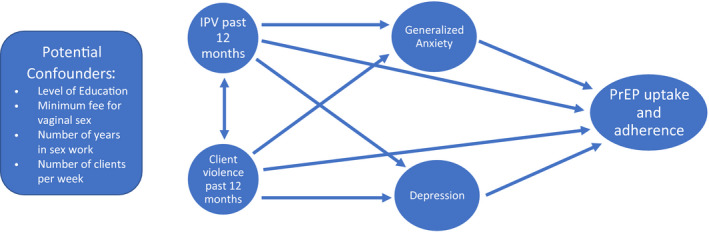
Model of associations tested. Conceptual model of the associations between gender‐based violence, mental health symptomatology, and use of PrEP medication. IPV, intimate partner violence; PrEP, pre‐exposure prohylaxis

The Sex Worker Outreach Programme (SWOP) in Nairobi, Kenya has been operating for over 20 years to provide HIV prevention, care and treatment to key populations in Nairobi county, with over 42,000 FSW enrolled as of 2019. Seven clinics across the county provide free access to HIV testing and treatment at every visit (including PrEP to all HIV‐negative FSW), reproductive healthcare services (including free condoms) and support for women who experience partner violence. Therefore, the SWOP provides the ideal opportunity to explore the associations between gender‐based violence against FSW, mental health outcomes and the use of PrEP medication. We aimed to: (i) determine the differential associations of IPV and client‐perpetrated violence with mental health outcomes; (ii) determine the potential effects of IPV and client‐perpetrated violence on the uptake of PrEP and (iii) determine the potential role of mental health symptomatology in PrEP uptake. Specifically, we hypothesize: (i) greater IPV and client‐perpetrated violence of any type will be associated with higher depressive and generalized anxiety symptoms, (ii) greater IPV and client‐perpetrated physical and sexual violence will increase PrEP use, whereas greater emotional violence will decrease PrEP use and (iii) higher depressive and generalized anxiety symptoms will be associated with a reduction in PrEP use (Figure 1).

## 2 Methods

### 2.1 Participant recruitment and survey administration

An anonymous questionnaire was administered to Nairobi FSW attending Kenya Aids Control Project (KACP) clinics during June‐July 2019. All women who were currently exchanging sex for money or goods were considered FSW for the purposes of this study. Participants were eligible if they were HIV‐negative and over the age of 18. Participants were recruited on a convenience basis across the seven clinics. Participation was voluntary, informed consent was provided and participants were compensated 300 KSH. The study was part of a quality improvement initiative approved by the Institutional Review Boards at Kenyatta National Hospital (Kenya) and the Universities of Toronto and Manitoba (Canada). Surveys were administered in a one‐on‐one interview in Kiswahili or English, and responses were recorded by staff administering the survey. HIV testing was performed according to Kenyan national guidelines, with initial screening by antibody‐based rapid test Determine HIV 1/2 (Inverness Medical, Tokyo, Japan) and confirmation of positive tests using SD Bioline HIV 1/2 (Standard Diagnostics Inc., Kyonggi Do, South Korea).

### 2.2 Measures of intimate partner, client‐perpetrated and other violence

An intimate partner was defined as any non‐paying partner, such as a husband or boyfriend. A client was defined as a paying partner (money, rent, school fees, etc.). IPV and client‐perpetrated violence was defined as any violence perpetrated by intimate partners or clients against the women and manifested through acts of physical, sexual or emotional violence. Other perpetrators were defined as anyone other than an intimate partner or client (e.g. police, city askaris, family members) who also committed the aforementioned acts of violence. The items were structured using a modified version of the World Health Organization Violence Against Women Instrument (VAWI), which assessed experiences of 13 specific acts of physical (six items), sexual (three items) or emotional violence (four items) from regular partners such as husbands or boyfriends [26]. An extra item assessing forced sex without a condom was added to the sexual violence section, for a total of 14 items. A “yes” to at least one question in each category constituted an experience of violence, and women were dichotomized accordingly in each violence sub‐group (physical, sexual, emotional). The items were asked once for perpetration of violence by an intimate partner (husband or boyfriend), once for the perpetration of violence by clients, and once for any other perpetration of violence. An overall variable was also created to represent any violence, which included any form (emotional, physical, sexual) perpetrated by any of an intimate partner, client or other in the past 12 months. The VAWI has demonstrated good internal validity (Cronbach’s α = 0.88) [27].

### 2.3 PrEP medication use

PrEP use was operationalized into four categories determined by the following question series. Participants were asked “Have you ever used PrEP?” If no, they were categorized as “never used.” If yes, participants were asked “Are you currently taking PrEP?” If no, they were categorized as a “past user.” If yes, participants were asked “How often do you take your PrEP pill?” If participants responded with less than six to seven times per week, they were categorized as a “current sub‐optimal user”. If participants responded six to seven times per week they were catergorized as a “current optimal user.”

### 2.4 Depressive symptoms

Current levels of depressive symptoms were assessed using the nine‐item self‐reported Patient Health Questionnaire‐9 (PHQ‐9) to assess both diagnostic categories and severity of symptoms [20]. Participants rate the chronicity of symptoms using a four‐point scale ranging from 0 (Not at all) to 3 (Nearly every day). Total scores of all items were summed, and participants were categorized as meeting criteria for moderate depression (PHQ‐9 ≥ 10) or not meeting criteria (PHQ‐9 < 10). The PHQ‐9 has been utilized widely in both research and clinical settings and possesses strong psychometric properties [28, 29]. Two large‐scale validation studies in healthcare settings found excellent internal consistency for the measure (Cronbach’s α = 0.86 to 0.89) and support for strong test–retest reliability (r = 0.84) across a 48‐h timeframe [30]. It has been validated among Kenyan HIV/AIDS populations (α = 0.78), with acceptable test‐retest reliability (ICC = 0.59) [31].

### 2.5 Generalized anxiety symptoms

Current levels of anxiety symptoms were similarly measured using the seven‐item self‐reported Generalized Anxiety Disorder‐7 (GAD‐7) designed to assess for both presence and severity of symptoms of generalized anxiety disorder [32]. Participants rate the chronicity of symptoms using a four‐point scale ranging from 0 (Not at all) to 3 (Nearly every day). Total scores of all items were summed, and participants were categorized as meeting criteria for moderate generalized anxiety (GAD ≥ 10) or not meeting criteria (GAD<10). The GAD‐7 has demonstrated strong psychometric properties in validation studies including excellent internal consistency (Cronbach’s α = 0.92) and strong test–retest reliability (r = 0.83). It has been validated among Kenyan HIV/AIDS populations (α = 0.82), with acceptable test–retest reliability (ICC = 0.70) [33].

### 2.6 Statistical analysis

Categorical variables were reported as counts with percentages and analysed with chi‐square or Fisher’s exact test whenever appropriate. Dichotomous variables were reported with percentages. Continuous variables were assessed for normal distribution using a normal probability plot, and were reported as mean with standard deviation if normally distributed or median with interquartile range (IQR) if not normally distributed. Continuous variables were compared between groups using Welch’s t‐test if normally distributed or Mann–Whitney U test if not normally distributed. There were no multiple imputations performed. Data were 99.9% complete.

The following three outcomes were evaluated with multivariable logistic regression models: depression (PHQ9 ≥ 10), anxiety (GAD7 ≥ 10) and use of PrEP (current use vs. past or no use). The following a priori covariates (chosen based on clinical relevance and parsimony) were included in the models: emotional IPV, physical IPV, sexual IPV, client emotional violence, client physical violence, client sexual violence, other emotional violence, other physical violence, other sexual violence, any violence, level of education (primary education or less, secondary education, postsecondary education), minimum fee for vaginal sex, number of years in sex work and number of clients per week. Participants with no intimate partner were excluded from multivariate models where IPV was a covariate. Measures of violence may be collinear, so we calculated the variance inflation factor for all multivariable models; all were <4. Beta‐coefficients were exponentiated for clinical interpretability. Generalized estimating equations were used to assess for clustering by site, which was non‐contributory. If using a Bonferroni correction for the nine different types of violence predictors, the significance level would become (α** = **0.05/9 = 0.006). Statistical analysis was performed in R (version 3.6.2).

## 3 Results

### 3.1 Participant demographics and experiences of violence

In total, questionnaires were completed by 220 HIV‐negative clinic attendees meeting the study criteria (Table 1).

**Table 1:** Cohort demographics

Characteristic	Total = 220^a^
Age	32 (27 to 39)
Age started sex work	23 (19 to 29)
Years in sex work	7 (4 to 12)
Level of education	
Primary education or less	105 (47.7%)
Secondary education	89 (40.5%)
Postsecondary education	26 (11.8%)
Casual clients per week	4 (3 to 7)
Regular clients per week	5 (3 to 8)
Minimum fee for vaginal sex (KSH)	500 (200 to 925)
History of sexually transmitted infection	142 (64.8%)
IPV (past 12 months)	
Any IPV	129/164 (78.7%)
Emotional	109/164 (66.5%)
Physical	94/163 (57.7%)
Sexual	94/164 (57.3%)
Client violence (past 12 months)	
Any client violence	178 (80.9%)
Emotional	162 (73.6%)
Physical	115 (52.3%)
Sexual	151 (68.6%)
Other violence (past 12 months)	
Any other violence	109 (49.5%)
Emotional	108 (49.1%)
Physical	88 (40%)
Sexual	50 (22.7%)
Any violence overall (past 12 months)	198 (90.0%)
PHQ9	10 (8 to 14)
≥10	125 (56.8%)
GAD7	8 (6 to 13)
≥10	86 (39.1%)
PrEP use (*215)	
Current optimal user	91 (42.3%)
Current sub‐optimal user	9 (4.2%)
Never used	84 (39.1%)
Past user	31 (14.4%)

Continuous variables are reported as median (interquartile range) unless otherwise specified. GAD7, Generalized Anxiety Disorder‐7; IPV, intimate partner violence; KSH, Kenyan Shilling; PHQ9, Patient Health Questionnaire‐9; PrEP, pre‐exposure prophylaxis.

^a^ Denominator is 220 unless otherwise specified.

The prevalence of probable depression was 56.8% (125/220). The prevalence of probable anxiety was 39.1% (86/220). Overall, 90% (198/220) of women reported a history of any violence in the past 12 months. 80.9% (178/220) reported client‐perpetrated violence. When stratifying violence types, 73.6% (162/220) reported client‐perpetrated emotional violence, 52.3% (115/220) reported client‐perpetrated physical violence and 68.6% (151/220) reported client‐perpetrated sexual violence. Of 164 women with an intimate partner, 78.7% (129/164) reported IPV in the past 12 months. 66.5% (109/164) reported emotional IPV, 57.7% (94/163, 1 missing data) reported physical IPV and 57.3% (94/163) reported sexual IPV. There were 49.5% (109/220) of women who reported violence by other perpetrators in the past 12 months. Of the 220 total participants, 42.3% (91/215, 5 missing data) were categorized as current optimal PrEP users.

### 3.2 Recent experience of violence was associated with depression and anxiety

To explore the associations of violence with mental health, total depressive and generalized anxiety scores were first compared between participants who had and had not experienced IPV (emotional, physical and/or sexual). Overall, participants who reported having experienced any of these forms of IPV in the past 12 months reported significantly higher depression and generalized anxiety scores than those who did not (Table 2).

**Table 2:** Demographics of FSW who experienced any IPV in the last 12 months versus FSW who have not experienced any IPV in the last 12 months

Characteristic	No recent IPV history (n = 35)	Recent IPV history (n = 129)	*p*‐value
Age	32 (26.5 to 38.5)	31 (26 to 37)	0.371
Age started sex work	25 (22 to 30)	20 (18 to 26)	<0.001
Years in sex work	6 (3 to 9.5)	8 (4 to 13)	0.053
Level of education			
Primary education or less	17 (48.6%)	57 (44.2%)	0.211
Secondary education	11 (31.4%)	58 (45%)	
Postsecondary education	7 (20%)	14 (10.9%)	
Casual clients per week	4 (3 to 5.5)	4 (3 to 7)	0.382
Regular clients per week	5 (3.5 to 6)	5 (3 to 9)	0.947
Minimum fee for vaginal sex (KSH)	500 (300 to 1000)	500 (300 to 900)	0.654
History of sexually transmitted infection	16 (47.1%)	85 (65.9%)	0.07
Client violence (past 12 months)			
Any client violence	16 (45.7%)	113 (87.6%)	<0.001
Emotional	14 (40%)	100 (77.5%)	<0.001
Physical	8 (22.9%)	72 (55.8%)	0.001
Sexual	12 (34.3%)	98 (76%)	<0.001
Other violence (past 12 months)			
Any other violence	13 (37.1%)	66 (51.2%)	0.2
Emotional	12 (34.3%)	66 (51.2%)	0.114
Physical	9 (25.7%)	51 (39.5%)	0.191
Sexual	3 (8.6%)	31 (24%)	0.077
PHQ9	7 (5 to 10.50)	12 (9 to 14)	<0.001
≥10	11 (31.4%)	84 (65.1%)	0.001
GAD7	5 (3 to 9.5)	9 (6 to 13)	0.001
≥10	9 (25.7%)	55 (42.6%)	0.104
PrEP use			
Current optimal user	12 (35.3%)	55 (43.3%)	0.198
Current sub‐optimal user	2 (5.9%)	5 (3.9%)	
Never used	18 (52.9%)	46 (36.2%)	
Past user	2 (5.9%)	21 (16.5%)	

Continuous variables are reported as median (interquartile range) unless otherwise specified. Continuous variables evaluated with Welch’s t‐test if normally distributed or Mann–Whitney U test if non‐normally distributed. Categorical variables evaluated with chi‐square or Fisher’s exact test whenever appropriate. FSW, female sex workers; IPV, intimate partner violence; PHQ9, Patient Health Questionnaire‐9; GAD7, Generalized Anxiety Disorder‐7; PrEP, pre‐exposure prophylaxis; KSH, Kenyan Shilling.

A similar analysis was then carried out to assess the association of client‐perpetrated violence (emotional, physical and/or sexual) with mental health, and recent client‐perpetrated violence was again associated with higher depression and generalized anxiety scores (Table 3).

**Table 3:** Demographics of FSW based on the experience of any client‐perpetrated violence in the last 12 months

Characteristic	No recent client violence history (n = 42)	Recent client violence history (n = 178)	*p*‐value
Age	32 (26.25 to 38.75)	32 (27 to 39)	0.759
Age started sex work	24 (20 to 30)	23 (18.25 to 28)	0.156
Years in sex work	6.5 (2 to 10)	7 (4 to 13)	0.147
Level of education			
Primary education or less	19 (45.2%)	86 (48.3%)	0.846
Secondary education	17 (40.5%)	72 (40.4%)	
Postsecondary education	6 (14.3%)	20 (11.2%)	
Casual clients per week	4 (3 to 5)	4 (3 to 7.75)	0.304
Regular clients per week	4.5 (3 to 6.75)	5 (3 to 8)	0.552
Minimum fee for vaginal sex (KSH)	500 (300 to 1000)	500 (200 to 500)	0.155
History of sexually transmitted infection	16 (39%)	126 (70.8%)	<0.001
IPV (past 12 months)			
Any IPV (past 12 months)	16 (45.7%)	113 (87.6%)	<0.001
Emotional	11 (31.4%)	98 (76%)	<0.001
Physical	10 (28.6%)	84 (65.6%)	<0.001
Sexual	6 (17.1%)	88 (68.2%)	<0.001
Other violence (past 12 months)			
Any other violence	6 (14.3%)	103 (57.9%)	<0.001
Emotional	5 (11.9%)	103 (57.9%)	<0.001
Physical	5 (11.9%)	83 (46.6%)	<0.001
Sexual	3 (7.1%)	47 (26.4)	0.013
PHQ9	7.5 (5 to 11.75)	11 (9 to 14)	<0.001
≥10	14 (33.3%)	111 (62.4%)	0.001
GAD7	5.5 (3 to 8.75)	9 (6 to 13)	<0.001
≥10	9 (21.4%)	77 (43.3%)	0.015
PrEP use			0.338
Current optimal user	17 (41.5%)	74 (42.5%)	
Current sub‐optimal user	1 (2.4%)	8 (4.6%)	
Never used	20 (48.8%)	64 (36.8%)	
Past user	3 (7.3%)	28 (16.1%)	

Continuous variables are reported as median (interquartile range) unless otherwise specified. Continuous variables evaluated with Welch’s t‐test if normally distributed or Mann–Whitney U test if non‐normally distributed. Categorical variables evaluated with chi‐square or Fisher’s exact test whenever appropriate. FSW, female sex workers; GAD7, Generalized Anxiety Disorder‐7; IPV, intimate partner violence; KSH, Kenyan Shilling; PHQ9, Patient Health Questionnaire‐9; PrEP, pre‐exposure prophylaxis.

Univariable and multivariable logistic regression models examining associations of violence with depression are presented in Table 4. Overall, any emotional IPV within the last 12 months and a secondary school education were each associated with an increased likelihood of probable depression (PHQ9≥10).

**Table 4:** Univariable and multivariable logistic regression models of probable depression among FSW

	Univariable		Multivariable v1		Multivariable v2	
			n = 163		n = 220	
	OR (95% CI)	*p*	OR (95% CI)	*p*	OR (95% CI)	*p*
IPV emotional	7.36 (3.55, 15.3)	<0.0001	7.52 (2.76, 20.5)	<0.0001		
IPV physical	2.49 (1.31, 4.73)	<0.01	0.71 (0.28, 1.82)	0.48		
IPV sexual	2.41 (1.28, 4.56)	<0.01	0.83 (0.32, 2.18)	0.7		
Client emotional violence	3.5 (1.86, 6.57)	<0.0001	1.39 (0.46, 4.18)	0.56		
Client physical violence	2.41 (1.39, 4.16)	<0.01	1.95 (0.73, 5.2)	0.18		
Client sexual violence	2.21 (1.24, 3.94)	<0.01	0.87 (0.3, 2.47)	0.79		
Other emotional violence	2.41 (1.39, 4.16)	<0.01	1.25 (0.37, 4.29)	0.72		
Other physical violence	2.4 (1.36, 4.23)	<0.01	1.23 (0.34, 4.52)	0.75		
Other sexual violence	3.46 (1.66, 7.21)	<0.001	2.24 (0.62, 8.07)	0.22		
Secondary education	3.11 (1.71, 5.65)	<0.001	3.86 (1.63, 9.16)	<0.01	3.41 (1.81, 6.44)	<0.001
Postsecondary education	2.05 (0.85, 4.94)	0.11	1.48 (0.4, 5.53)	0.56	2.01 (0.73, 5.54)	0.18
Minimum fee vaginal sex (per 100 KSH)	1.02 (0.99, 1.06)	0.17	1.01 (0.97, 1.05)	0.77	1.02 (0.98, 1.06)	0.34
Years in sex work	0.99 (0.95, 1.03)	0.68	0.99 (0.93, 1.06)	0.76	0.99 (0.95, 1.04)	0.71
Casual clients per week	0.98 (0.93, 1.03)	0.35	0.96 (0.89, 1.02)	0.17	0.98 (0.93, 1.03)	0.38
Any violence	7.07 (2.31, 21.7)	<0.001			9.39 (2.9, 30.4)	<0.001

FSW, female sex workers; IPV, intimate partner violence; KSH, Kenyan Shilling.

Univariable and multivariable logistic regression models examining associations of violence with anxiety were then assessed, and results are presented in Table 5. Overall, any emotional IPV within the last 12 months and a lower fee for sex work were associated with an increased likelihood of probable anxiety (GAD7 ≥ 10).

**Table 5:** Univariable and multivariable logistic regression models of probable anxiety among FSW

	Univariable		Multivariable v1		Multivariable v2	
			n = 163		n = 220	
	OR (95% CI)	*p*	OR (95% CI)	*p*	OR (95% CI)	*p*
IPV emotional	3.27 (1.56, 6.87)	<0.01	3.61 (1.33, 9.76)	0.01		
IPV physical	1.48 (0.78, 2.83)	0.23	0.72 (0.3, 1.71)	0.46		
IPV sexual	1.97 (1.02, 3.78)	0.04	1.21 (0.49, 2.96)	0.68		
Client emotional violence	2.24 (1.15, 4.35)	0.02	1.67 (0.58, 4.83)	0.34		
Client physical violence	1.47 (0.85, 2.55)	0.16	0.88 (0.36, 2.17)	0.78		
Client sexual violence	1.73 (0.94, 3.17)	0.08	0.73 (0.26, 2.01)	0.54		
Other emotional violence	2.13 (1.23, 3.7)	<0.01	0.57 (0.17, 1.91)	0.36		
Other physical violence	2.52 (1.44, 4.41)	<0.01	3.4 (0.97, 12)	0.06		
Other sexual violence	2.46 (1.29, 4.67)	<0.01	1.25 (0.42, 3.66)	0.69		
Secondary education	2.04 (1.13, 3.67)	0.02	1.53 (0.72, 3.29)	0.27	2.21 (1.21, 4.04)	<0.01
Postsecondary education	1.96 (0.81, 4.69)	0.13	3.58 (0.99, 12.9)	0.05	3.12 (1.16, 8.44)	0.02
Minimum fee vaginal sex (per 100 KSH)	0.98 (0.95, 1.01)	0.26	0.95 (0.91, 1)	0.04	0.97 (0.93, 1.01)	0.09
Years in sex work	1 (0.97, 1.05)	0.82	0.98 (0.92, 1.04)	0.49	1 (0.96, 1.04)	0.95
Casual clients per week	1 (0.95, 1.05)	0.88	0.98 (0.92, 1.04)	0.48	0.99 (0.94, 1.04)	0.62
Any violence	3.18 (1.04, 9.75)	0.04			3.47 (1.1, 10.9)	0.03

FSW, female sex workers; IPV, intimate partner violence; KSH, Kenyan Shilling.

### 3.3 Associations of PrEP use with partner violence and mental health

PrEP use was first assessed based on the experience of IPV in the past 12 months. While there was no association of PrEP with a recent experience of emotional IPV (Pearson χ^2^ = 0.63; *p* = 0.43) or physical IPV (Pearson χ^2^ = 1.32, *p* = 0.25), participants who had experienced sexual IPV were more likely to be taking PrEP (Pearson χ^2^ = 6.08, *p* = 0.014). While no univariable associations were found between PrEP use and emotional, physical or sexual violence initiated by casual clients (Pearson χ^2^ = 0.02, 1.14 and 2.32 respectively; all *p* > 0.05), subsequent multivariable logistic regression found that client‐perpetrated physical violence was independently linked with PrEP use, and client‐perpetrated emotional violence was independently linked with a lower likelihood of PrEP use (Table 6).

**Table 6:** Univariable and multivariable logistic regression models of current PrEP use among FSW

	Univariable		Multivariable v1		Multivariable v2	
			n = 160		n = 160	
	OR (95% CI)	*p*	OR (95% CI)	*p*	OR (95% CI)	*p*
IPV emotional	1.31 (0.67, 2.54)	0.43	0.95 (0.38, 2.36)	0.9	1.2 (0.45, 3.21)	0.71
IPV physical	1.45 (0.77, 2.74)	0.25	1.31 (0.57, 3.04)	0.52	1.29 (0.55, 3)	0.55
IPV sexual	2.23 (1.17, 4.25)	0.01	1.99 (0.82, 4.82)	0.13	2.03 (0.83, 4.96)	0.12
Client emotional violence	0.95 (0.52, 1.75)	0.88	0.23 (0.07, 0.71)	0.01	0.24 (0.08, 0.74)	0.01
Client physical violence	1.34 (0.78, 2.3)	0.29	3.01 (1.16, 7.81)	0.02	3.2 (1.22, 8.41)	0.02
Client sexual violence	1.58 (0.88, 2.84)	0.13	1.75 (0.64, 4.81)	0.28	1.7 (0.62, 4.7)	0.31
Other emotional violence	1.27 (0.74, 2.18)	0.38	0.45 (0.14, 1.48)	0.19	0.44 (0.13, 1.46)	0.18
Other physical violence	1.54 (0.89, 2.66)	0.12	2.49 (0.73, 8.48)	0.15	2.7 (0.78, 9.35)	0.12
Other sexual violence	1.64 (0.87, 3.11)	0.13	1.54 (0.51, 4.65)	0.44	1.7 (0.55, 5.18)	0.35
Secondary education	0.97 (0.55, 1.72)	0.91	1 (0.47, 2.12)	0.99	1.16 (0.53, 2.55)	0.71
Postsecondary education	1.52 (0.63, 3.66)	0.35	2.45 (0.68, 8.79)	0.17	2.7 (0.74, 9.8)	0.13
Minimum fee vaginal sex (per 100)	0.98 (0.95, 1.01)	0.19	0.97 (0.93, 1)	0.08	0.96 (0.93, 1)	0.08
Years in sex work	1.02 (0.98, 1.06)	0.42	0.95 (0.9, 1.01)	0.08	0.95 (0.89, 1)	0.06
Casual clients per week	1 (0.95, 1.05)	0.99	0.97 (0.91, 1.03)	0.32	0.96 (0.9, 1.02)	0.23
Depressive symptoms	0.67 (0.39, 1.16)	0.15			0.62 (0.25, 1.55)	0.3
Anxiety symptoms	0.82 (0.47, 1.42)	0.48			0.78 (0.33, 1.84)	0.58

FSW, female sex workers; IPV, intimate partner violence; KSH, Kenyan Shilling.

Next, we assessed associations of PrEP use with mental health based on total depressive and generalized anxiety scores. Depressive symptomatology did not differ between PrEP users ( $\bar{x}$ = 10.48) and non‐users ( $\bar{x}$ = 11.38), nor did anxiety scores between PrEP users ( $\bar{x}$ = 8.69) and non‐users ( $\bar{x}$ = 9.10), (both *p* > 0.05). Neither depression or anxiety significantly predicted PrEP use in subsequent multivariable logistic regression (Table 6).

## 4 Discussion

There is high HIV incidence and prevalence among FSW in sub‐Saharan Africa [2], and PrEP is a highly effective HIV prevention tool [9, 10]. However, the uptake of PrEP within these communities has been suboptimal [34]. Given the prior linkage of violence with both HIV acquisition and adverse mental health outcomes, the aim of our study was to explore the associations between specific types of IPV and client‐perpetrated violence, mental health outcomes, and the uptake of effective HIV prevention services (PrEP) among FSW from Nairobi, Kenya. Our goal was to identify barriers to effective HIV prevention that may constitute targets for future interventions. We found that emotional, physical and sexual violence were very common among Nairobi FSW; participants who had experienced any of these forms of violence, regardless of the perpetrator, were more likely to experience depressive and generalized anxiety symptoms, although the strongest associations were with emotional IPV. A history of sexual IPV (but not other forms of violence) was associated with enhanced PrEP uptake; furthermore, client‐perpetrated physical violence was linked to increased PrEP use, whereas client‐perpetrated emotional violence was associated with decreased PrEP use. These findings suggest that interventions to reduce gender‐based violence may independently enhance HIV prevention and reduce the mental health burden in this community.

The fact that FSW who used PrEP were more likely to have experienced sexual IPV, and that client‐perpetrated physical violence was associated with increased PrEP use, is in keeping with a systematic review by Mugo and colleagues [22] who found that PrEP uptake, adherence and retention in Africa is enhanced in persons who perceive themselves to be at high risk for HIV infection. Clearly, women who have experienced sexual IPV may feel particularly vulnerable, given that sex is the most common route of HIV transmission [2], and FSW find it more difficult to use male condoms with their intimate partners compared to casual clients [35]. Indeed, it is likely due to these barriers that intimate partners have been found to contribute more to HIV transmission in FSW communities than casual clients [35, 36]. Our results support independent modelling studies which suggested that the elimination of sexual violence alone would avert 17% of HIV infections in Kenya among FSW and their clients in the next decade [37]. Furthermore, client‐perpetrated emotional violence in our community was independently linked to decreased PrEP use, supporting literature which suggest that different patterns of violence among FSW in Kenya are associated with distinct sexual risk outcomes [21]. Together, these findings have important implications for strategies aiming to reduce gender‐based violence against FSW, not only as a fundamental human right but also to reduce the community spread of HIV/AIDS.

In our study, women who experienced intimate partner or client‐perpetrated emotional, physical or sexual violence displayed higher levels of depressive and generalized anxiety symptoms. This is in accordance with a recent study by Roberts and colleagues [21] in Mombasa, Kenya which found that women with severe gender‐based violence had higher scores for depressive symptoms, post‐traumatic stress disorder symptoms and disordered alcohol use, and concluded that PrEP would be an important HIV prevention tool in the community. While our own study found mental health to be more strongly linked with emotional IPV specifically, other recent research also suggests that emotional IPV may be a particularly important contributor to adverse mental health outcomes [38]. This may be especially true in FSW who often depend on their intimate partners for basic survival needs [39]. Our study extends these findings by defining specific associations between mental health and both perpetrator and violence type, and by assessing associations of generalized anxiety symptoms, which were also extremely prevalent among our participants.

Although our study did not find depression or anxiety to be associated with PrEP use, mental health concerns were still very prevalent in this community. Others have demonstrated that depression, but not anxiety, decreased antiretroviral treatment adherence among women living with HIV [40], but our findings are important in the context of antiretrovirals for prevention, particularly given the very high rates of depression and generalized anxiety in this sex worker community. While interventions exist that focus on integrating culturally sensitive mental health services into Kenyan communities [41, 42, 43], these services may be failing to reach key vulnerable populations. A recent systematic review and meta‐analysis on mental health in FSW in low‐ and middle‐income countries did not find any single intervention that was designed to address mental disorders among FSW [16]. Research in this field is urgently needed in order to provide effective evidence‐based mental healthcare to this key population. Specific programmes have been shown to be effective in reducing violence against women in Uganda and Tanzania [44, 45], as well as violence against FSW in South India, and these may serve as the basis for developing programmes targeting FSW in Kenya [46]. In addition, existing programmes in Nairobi, Kenya that have demonstrated success in reducing violence against girls through empowerment, such as the IMPower/SOS programme, could be adapted for use in FSW populations [47].

Despite these important findings, our study has potential limitations. Questionnaires were only administered to FSW attending KACP clinics, and data could only be assessed from those attendees who agreed to participate. Therefore, it remains unknown to what extent the results apply to FSW not in care, who accessed other services, or who declined the questionnaire. In addition, the use of a facility‐based convenience sample may have led to our study having an enriched enrolment of FSW taking PrEP. Our study is cross‐sectional in design, and so the direction of causation cannot be defined for the associations that we describe. Furthermore, reporting bias (overreporting of PrEP and/or underreporting of violence) may have skewed associations. Our results may have been confounded by other unmeasured factors, such as adverse childhood experiences, violence predating the 12‐month time frame, indirect cost of accessing services, or other non‐measured reasons for non‐adherence to PrEP such as fear of side effects. Nonetheless, our results have clear implications for quality improvement within the programme and merit broader consideration within female sex worker clinics elsewhere.

## 5 Conclusions

In summary, this study demonstrates that among FSW attending KACP clinics in Nairobi, Kenya, those who experience sexual IPV were more likely to use PrEP, whereas client‐perpetrated violence differentially affected the use of PrEP medication. Women who experienced any form of emotional, physical or sexual violence currently had greater symptomatology for depression and generalized anxiety, with emotional IPV particularly associated with mental health symptomatology. These findings stress the importance of developing targeted strategies aimed at addressing gender‐based violence for FSW, while also providing mental health support services particularly to women who have suffered from these abuses.

## Competing interests

The authors declare no conflicts of interest exist.

## Authors’ contributions

ML, MM, TB, RK and JK designed and conceived the study. AK^1^ and LS performed the data analyses. ML and AK^1^ wrote the manuscript. ML, MM, AK^2^ and JK coordinated data collection and management. All authors critically reviewed, edited and approved the final manuscript.

## Abbreviations

FSW, female sex workers; GAD7, Generalized Anxiety Disorder‐7; IPV, intimate partner violence; KACP, Kenya Aids Control Project; PHQ9, Patient Health Questionnaire‐9; PrEP, pre‐exposure prophylaxis; VAWI, Violence Against Women Instrument.
